# Ultrasound location of ground-glass opacity during thoracoscopic surgery

**DOI:** 10.1093/icvts/ivac234

**Published:** 2022-09-05

**Authors:** Gaetana Messina, Mary Bove, Giovanni Natale, Antonio Noro, Mario Martone, Giorgia Opromolla, Vincenzo Di Filippo, Beatrice Leonardi, Morena Fasano, Rita Polito, Alfonso Fiorelli, Mario Santini, Giovanni Vicidomini

**Affiliations:** Thoracic Surgery Unit, Università degli Studi della Campania “Luigi Vanvitelli”, Napoli, Campania, Italy; Thoracic Surgery Unit, Università degli Studi della Campania “Luigi Vanvitelli”, Napoli, Campania, Italy; Thoracic Surgery Unit, Università degli Studi della Campania “Luigi Vanvitelli”, Napoli, Campania, Italy; Thoracic Surgery Unit, Università degli Studi della Campania “Luigi Vanvitelli”, Napoli, Campania, Italy; Thoracic Surgery Unit, Università degli Studi della Campania “Luigi Vanvitelli”, Napoli, Campania, Italy; Thoracic Surgery Unit, Università degli Studi della Campania “Luigi Vanvitelli”, Napoli, Campania, Italy; Thoracic Surgery Unit, Università degli Studi della Campania “Luigi Vanvitelli”, Napoli, Campania, Italy; Thoracic Surgery Unit, Università degli Studi della Campania “Luigi Vanvitelli”, Napoli, Campania, Italy; Oncology, Department of Precision Medicine, Università della Campania “L. Vanvitelli”, Napoli, Campania, Italy; Nutrition Science, University of Foggia, Foggia, Italy; Thoracic Surgery Unit, Università degli Studi della Campania “Luigi Vanvitelli”, Napoli, Campania, Italy; Thoracic Surgery Unit, Università degli Studi della Campania “Luigi Vanvitelli”, Napoli, Campania, Italy; Thoracic Surgery Unit, Università degli Studi della Campania “Luigi Vanvitelli”, Napoli, Campania, Italy

**Keywords:** Ultrasound, Ground-glass opacity pattern, Thoracic surgery

## Abstract

**OBJECTIVES:**

Application of video-assisted thoracoscopy brought lung surgery into the minimally invasive era; the lack of tactile feedback using VATS, remains a disadvantage because surgeons are unable to locate lesions with a finger or device. This study aimed to investigate the effectiveness, the applicability and the utility of intraoperative ultrasound (IU), for the localization of small ground-glass opacity (GGO) lesions in the parenchyma, as a guide in finding their margins in a deflated lung.

**MATERIALS AND METHODS:**

We included 15 consecutive patients undergoing diagnostic resection of GGOs via VATS in the Thoracic Surgery Unit of the University of ‘Luigi Vanvitelli’ of Naples from November 2019 to December 2021. They were under general anaesthesia, when the lung had been collapsed, the probe was placed in the region where the target lesion was thought to reside on the basis of low-dose computed tomography scanning. GGO could be identified their sizes, echo levels and posterior echo was recorded by IU when the lung was completely deflated.

**RESULTS:**

We conducted a retrospective single-centre study. All GGOs were identified by IU. The mean size and depth were 14.1 ± 0.5 and 4.8 ± 0.3 mm, respectively. Six (40%) lesions had hyperechoic patterns, 9 (60%) had mixed echogenicity where the hyperechoic patterns were irregularly mixed with hypoechoic patterns. The final diagnoses included 2 (15%) atypical adenomatous hyperplasia; 2 (15%) adenocarcinomas in situ; 3 (23%) minimally invasive adenocarcinomas and 6 (46%) invasive adenocarcinomas.

**CONCLUSIONS:**

The results of our study showed that IU could safely and effectively detect GGOs.

**Figure:**
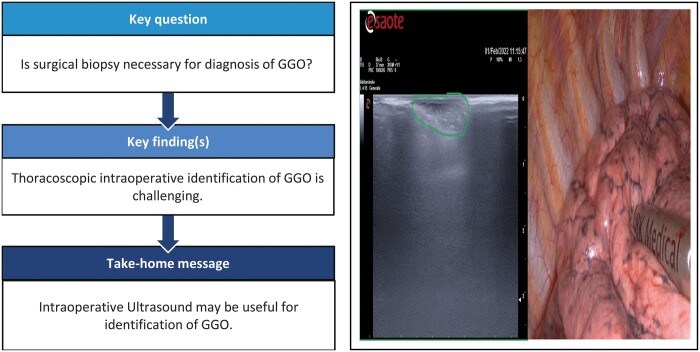

## INTRODUCTION

Over the past several decades, low-dose computed tomography has revolutionized screening worldwide of lung cancer, hence more and more people are being screened for lung cancer in anticipation of early diagnosis; therefore, small solid pulmonary nodules and ground-glass opacity (GGO) can be detected in time.

Along with the popularity of low-dose computed tomography screening, it is possible to increasingly detect small GGO and analyse its morphologic, anatomic and quantitative information [1–3]; although GGO is a non-specific radiologic finding, the persistent GGO is more likely to be malignant.

GGOs are defined as an area of hazy increased attenuation that does not obscure underlying bronchial structures or vascular, markings on high-resolution computed tomography. Since GGOs can be observed in both malignant and benign conditions, differential diagnosis is crucial to define a prompt treat [4].

GGO is classified into pure GGO and mixed GGO on high-resolution computed tomography, based on the absence or presence of solid component.

The widespread application of video-assisted thoracoscopy brought lung surgery into the minimally invasive era; the lack of tactile feedback while using VATS remains a major disadvantage because surgeons are unable to precisely locate lesions with a finger or device [5].

Pure ground-glass nodules [with computed tomography (CT) values less than −500 HU] are difficult to identify intraoperatively *in vivo*, and mixed ground-glass nodules (with CT values between −500 HU and −100 HU), are easily palpated [6].

Thus, the establishment of techniques that can confirm the detection of these tumours, in collapsed lungs during VATS, and assist surgeons in determining the margin of the resected lung from the GGO is very important.

This study therefore aimed to investigate the effectiveness, the applicability and the utility of ultrasound intraoperative (IU), for the localization of small GGO lesions deep in the parenchyma, defining the characteristics and it could serve as a guide, in finding the proper margin in a deflated lung.

## MATERIALS AND METHODS

### Ethics statement

The study was conducted in compliance with the principles of the Declaration of Helsinki and written informed consent was obtained from all participants during preoperative communication; the protocol was approved by the Ethics Committee of the University of ‘Luigi Vanvitelli’ of Naples (32655/2021).

The endpoints of the study were to evaluate the ability of IU to detect GGOs and define the depth and size of the lesions.

We included all consecutive patients undergoing diagnostic resection of GGOs via VATS in the Thoracic Surgery Unit of the University of ‘Luigi Vanvitelli’ of Naples from November 2019 to December 2022.

All patients were undergoing general anaesthesia using a double-lumen endotracheal tube.

Just the patient had been rotated into the lateral decubitus position, single-lung ventilation was initiated so that the lung would have completely collapsed, so the thoracoscope was inserted into the pleural space.

After thoracoscopic examination and ensuring that there were no pleural adhesions, we set up the probe used to perform the ultrasound. The pleural space was filled with 500 ml of warm saline, obtaining a better definition IU view, because warm saline contributes to increase the conductivity of the ultrasounds preventing massive reflection.

**Figure 1:**
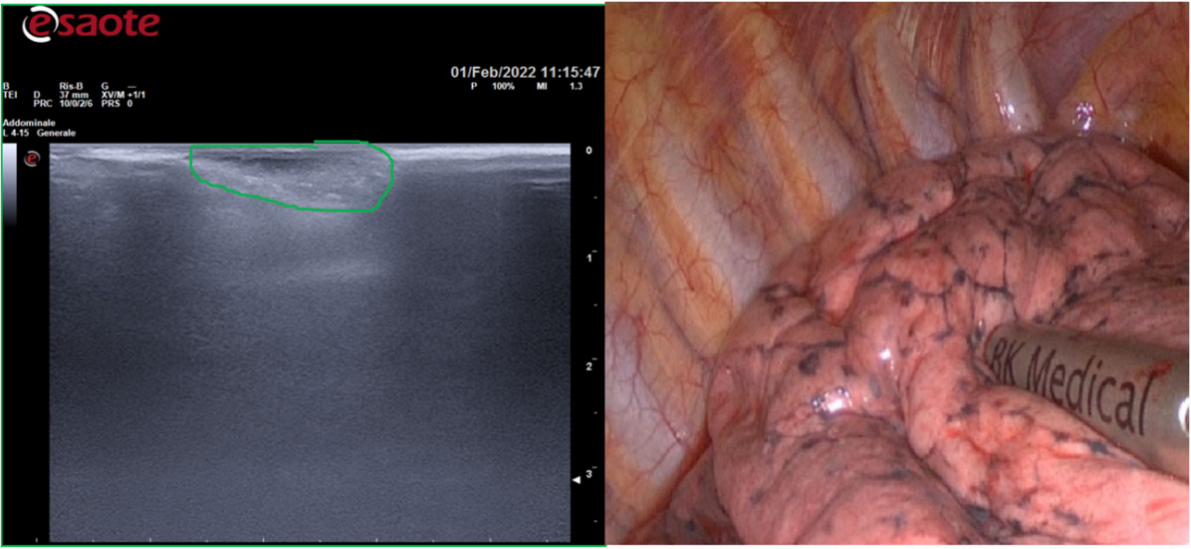
The probe was placed in the region of the target lesion. The ultrasound consents to distinguish the ground-glass opacity from the lung parenchyma.

When the lung had been collapsed completely, the probe was placed in the region of the target lesion where it was thought to reside on the basis of low-dose computed tomography scanning (Fig. 1).

If the lung could not be collapsed completely, gentle pressure to the lung surface with the IU probe was added to reduce residual air and careful suction of the airways was carried out.

**Figure 2:**
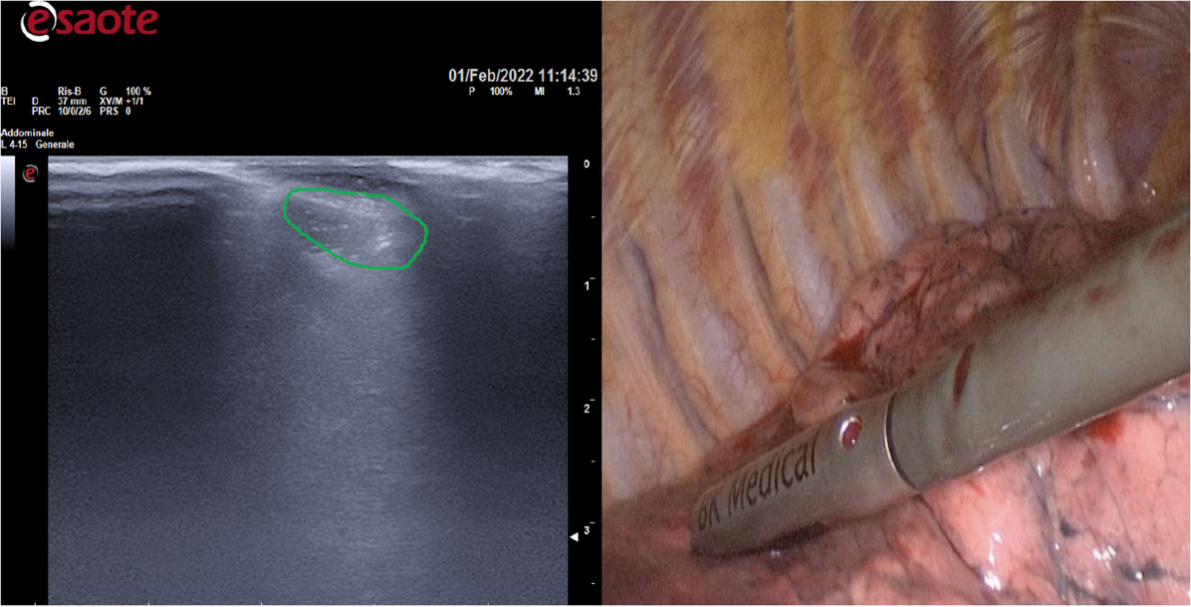
Intraoperative ultrasound can detect accurately the location and can define the characteristics of Ground-glass opacities.

GGO could be identified their sizes, echo levels and posterior echo was recorded by IU only when the lung was completely deflated (Fig. 2).

The ultrasound characteristics of GGOs were compared with those of the surrounding normal lung parenchyma. We used electrocautery to mark the visceral pleura to define the surgical margins of resection, considered as ‘adequate’ when the distance from the GGO was 1.5–2 cm to the resection.

### Ultrasound characteristics of ground-glass opacities

All pure GGOs (*n* = 6) presented hyperechoic patterns, whereas mixed GGOs (*n* = 9) had mixed either ultrasound patterns with both hyperechoic and hypoechoic components.

**Figure 3:**
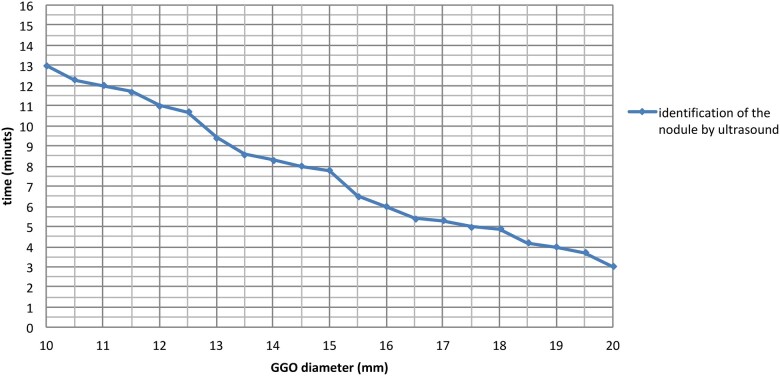
Lesion with a greater diameter was easier to find.

**Figure 4:**
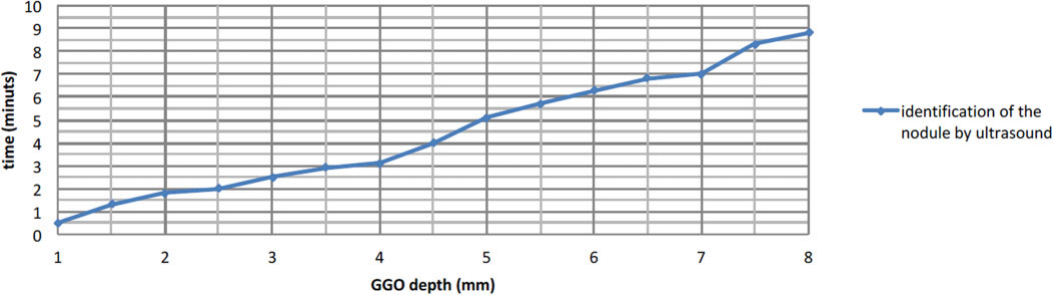
Lesion less deep from the visceral pleura was easier to find.

**Figure 5:**
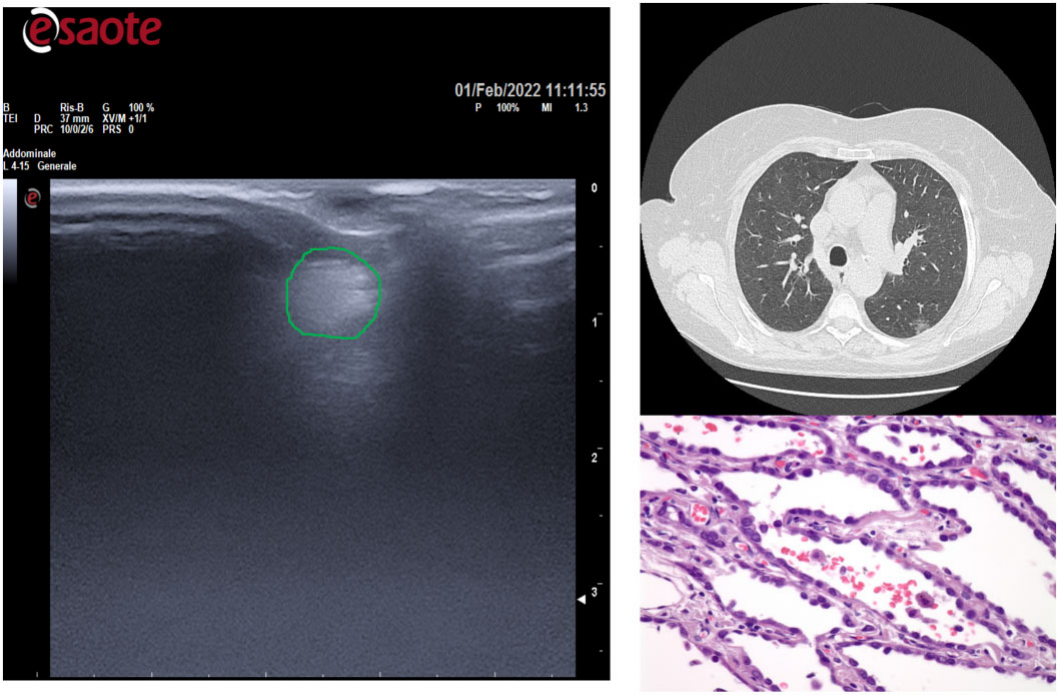
Atypical adenomatous hyperplasia.

Hyperechoic pattern predominated over the hypoechoic component in the pre-invasive lesions (*n* = 2; Fig. 3) and in the minimally invasive adenocarcinomas (*n* = 4; Fig. 4), whereas the hypoechoic component predominated in the invasive adenocarcinoma (*n* = 9; Fig. 5). However, IU is unable to differentiate histological subtypes of adenocarcinomas from the US patterns.

### Statistical analysis

In all cases, the pathologists guided by a landmark (e.g. stitches or clips) on the sample corresponding to the ultrasound identified GGO, easily found the lesion. The diagnosis was made at the first analysis, no additional sections or additional surgical biopsy was performed. Therefore, the Pearson correlation test was used to evaluate the correlation between the US and the histological measurements. A *P*-value measurement <0.05 was considered statistically significant. MedCalc statistical software (version 12.3; Broekstraat 52, 9030 Mariakerke, Belgium) was used for the analysis.

## RESULTS

We conducted an observational, retrospective, single-centre study in the Thoracic Surgery Unit of the University of ‘Luigi Vanvitelli’ of Naples from November 2019 to December 2021. We included all consecutive patients undergoing diagnostic resection of GGOs via VATS. During the study period, 31 patients were referred to our department because GGOs were detected on high-resolution CT scans performed for other clinical reasons. Of these, 18 GGOs, in agreement with the guidelines of the Fleischner Society [7] and of the Japanese Society [8], for management of incidental pulmonary nodules detected on CT images; patients with GGOs that had a solid component >5 mm (*n* = 11) or patients with GGOs >15 mm (*n* = 7) were suspected to be malignant.

In 3 of 18 (17%) cases, a diagnosis of poorly differentiated adenocarcinoma was made with a transcutaneous biopsy, and a lobectomy was performed immediately. The remaining 15 (83%) patients underwent a diagnostic resection of the GGOs via VATS.

The mean age of the study population was 57 years; 6 were women and 9 were men; respiratory and cardiac tests were within normal ranges, and no other preoperative comorbidities contraindicated an operation.

Inclusion criteria: respiratory and cardiac tests were within normal ranges, and no other preoperative comorbidities contraindicated an operation, absence of pleural adhesions. Since chest CT scan is moderately poor sensitive and specific for identification of pleural adhesions, in our centre, we perform transthoracic ultrasound to predict the presence of pleural adhesions. They can be identified with the absence of sliding lung sign [9] an echographic phenomenon produced during normal respiratory cycle, the visceral pleura sliding on the parietal pleura.

Exclusion criteria patients with history of asthma, pulmonary fibrosis, chronic obstructive pulmonary disease or severe heart disease, it is difficult to maintain single-lung ventilation during VATS.

Based on the CT consolidation, 6 lesions were classified as pure GGOs and 9 as mixed GGOs.

All GGOs were successfully identified by US, in about 12 min (9–13 min). The mean tumour size and depth were 14.1 ± 0.5 and 4.8 ± 0.3 mm, respectively: therefore, the more the lesion had a greater diameter and was less deep from the visceral pleura, the easier it was to find (Figs. 3 and 4).

Six (40%) lesions had hyperechoic patterns, while 9 (60%) lesions had mixed echogenicity where the hyperechoic patterns were irregularly mixed with hypoechoic patterns.

**Figure 6:**
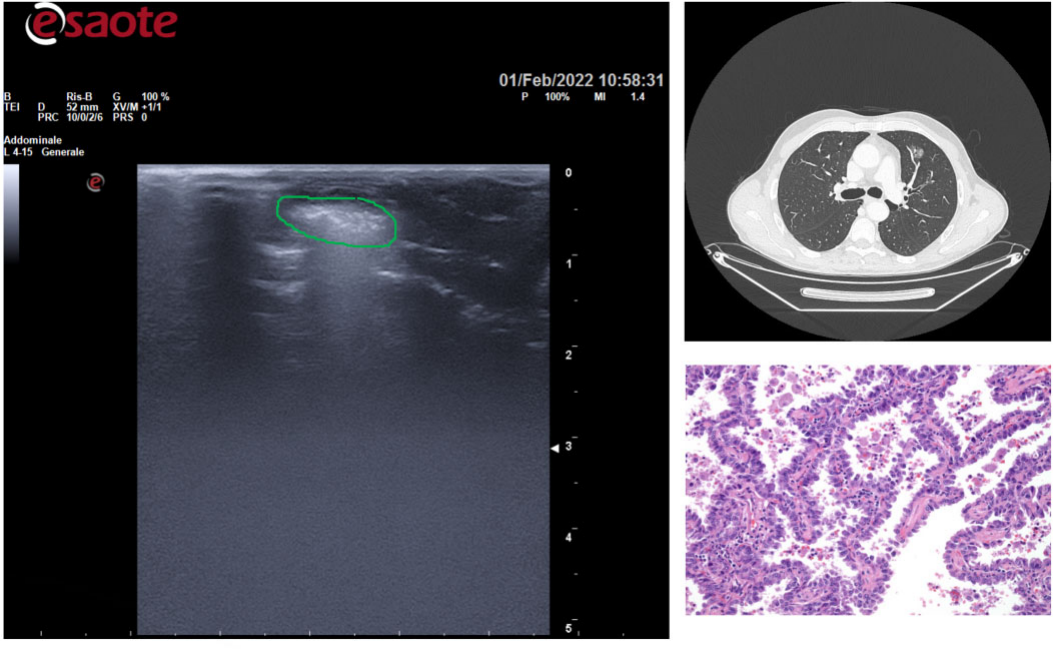
Lepidic adenocarcinoma.

**Figure 7:**
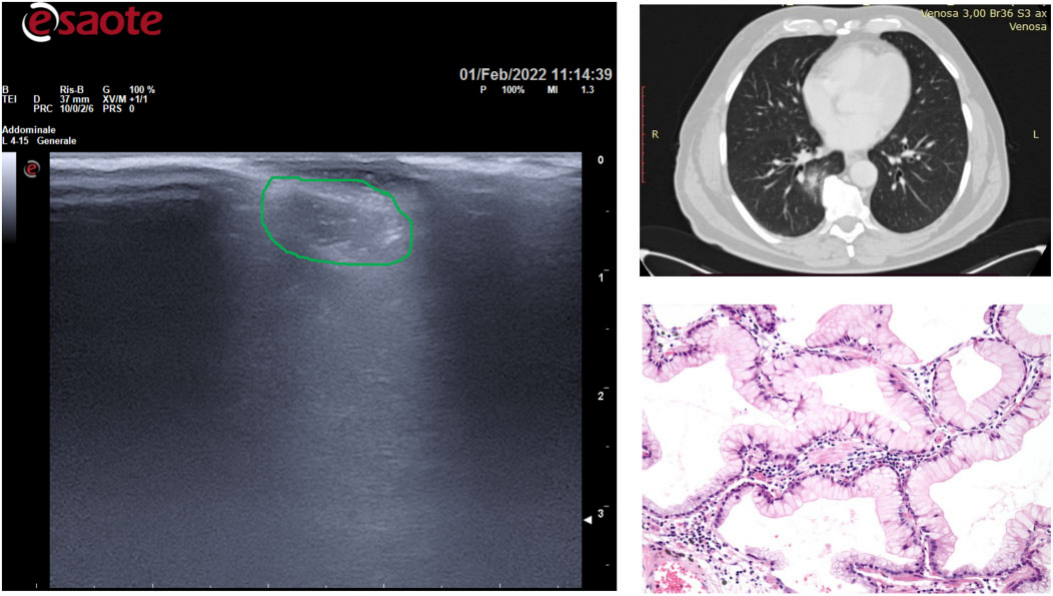
Invasive adenocarcinomas.

The final diagnoses included 2 (15%) atypical adenomatous hyperplasia (Fig. 5); 2 (15%) adenocarcinomas *in situ* (Fig. 6); 3 (23%) minimally invasive adenocarcinomas and 6 (46%) invasive adenocarcinomas (Fig. 7).

The Pearson correlation test was used to evaluate the correlation between the US and the histological measurements. A *P*-value measurement <0.05 was considered statistically significant.

A full collapsed lung could be achieved in 13 of these 15 cases; the ultrasound features did greatly correlate with a complete deflation of the lung. In all 13 patients in whom the complete collapse of the lung was achieved, the GGO produced high clear echo images. In patients in which the lung was not completely collapsed it was not possible to identify the lesion under ultrasound guidance. We used the anatomical landmarks with multiplanar CT reconstruction for the search of GGOs. However, the intervention was much longer for the search for GGOs.

The posterior ultrasound images of the GGO were found to be attenuated in 6 cases, accentuated in a further 7 lesions, but showed no change in 6 cases. Moreover, all acoustic shadows were attributed to high internal echo images, whereas all of the acoustic enhancements were ascribed to the presence of low internal echo images. The average maximum diameter of the GGO that had posterior echoes was particularly larger than the diameters of those that had no posterior echoes.

Based on the results of the frozen section analysis, diagnostic resection was followed: by wedge resection in 2 cases of atypical adenomatous hyperplasia, by segmentectomy in 5 cases (2 adenocarcinomas *in situ* and 3 minimally invasive adenocarcinomas) and by lobectomy in 6 cases of invasive adenocarcinomas. The characteristics of study population are shown in Table 1.

**Table 1:** The characteristics of study population

Patients, *n*	15
Pure GGO, *n* (%)	6 (33)
Mixed GGO, *n* (%)	9 (50)
Deflated lung, *n* (%)	13 (87)
Age, years (*mean*)	57
Sex (male), *n* (%)	9 (60)
Pure GGO size (mm) *mean*	14.1 ± 0.5
Mixed GGO size (mm) *mean*	4.8 ± 0.3
Distance from visceral pleura (cm)	1.5-2
Surgical operation, *n* (%)	
Wedge resection	2 (15)
Segmentectomy	5 (39)
Lobectomy	6 (46)
Histology, *n* (%)	
Atypical hyperplasia	2 (15)
Adenocarcinoma *in situ*	2 (15)
Minimally invasive adenocarcinoma	3 (23)
Invasive adenocarcinoma	6 (46)

GGO: ground-glass opacity.

In addition, the use of intraoperative ultrasonography did not prolong the operation time,

No complications were found from the use of IU in any of our 13 patients.

## DISCUSSION

Different preoperative techniques have the purpose of marking GGOs favouring its localization, such as indocyanine tattooing, microcoiling, hooking and radiolabelling [3, 10–12].

Though these methods have a success rate up to 100% and may avoid open thoracotomy in over 50% of cases, the complications as: pneumothorax, air embolism intraparenchymal and bleeding, is about 16.7%.

GGOs represent early lung cancer or precancerous lesions, where the likelihood of GGO malignancy can be as high as 59–73% [13–15]. The features of GGO tightly resemble the pulmonary parenchyma, for that reason, it is difficult to detect by finger palpation during surgery [16–19].

In thoracoscopic surgery, the surgery area for palpation is limited and if a subpleural sub-solid nodule is located at the back of the thorax or at a distance from the surgical incision, it will be difficult to palpate using fingers and sometimes can be impossible to locate, and consequently, it will result an increase in incisions, in the prolongation of surgery time, conversion to thoracotomy and even the expansion of resection [19–23].

Hence localization and complete resection of GGOs represent challenging tasks for thoracic surgeons.

In our study, we evaluated the ability of US to detect accurately the location and to define the characteristics of GGOs. The ultrasound was performed by an ultrasound-trained thoracic surgeon, the procedure is simple, but requires experience in ultrasound.

After bronchial exclusion, the lung completely collapses in about 90 s (70–110 s).

The selective intubation manoeuvre is not easy and requires experience and a lot of time in its implementation. The inadequate bronchial exclusion does not consent a full lung collapse. Also asthma, severe emphysema or fibrosis prevent the collapse of the whole lung.

However, if the lung had not been completely deflated, the structures of interior lungs were probably to display a ‘spotted hyperechoic pattern’ due to residual air echo artefacts; therefore, the presence of a GGO can be masked.

We devised a method for complete air removal from the airways and applying, with an instrumental probe, gentle pressure to the lung surface, to achieve a complete collapse of the lung.

However, we could not achieve a completely deflated lung in 17% of the patients, because of trapped sputum in the airway; thus, the collaboration of the anaesthetist is essential to maintain the lung in a completely deflated condition.

Therefore, complete lung collapse is essential for the reliability of this procedure.

This procedure has limitations in some patients in terms of its ability to detect their GGO, because asthma or severe emphysema have been shown errors in ultrasonographic evaluations and often it is difficult to maintain single-lung ventilation in patients with severe heart disease or pulmonary fibrosis.

All pure GGOs presented a hyperechoic pattern, and mixed GGOs had a mixed component with hyperechoic and hypoechoic patterns in a completely deflated lung.

Pure GGOs seen hyperechogenicity pattern, it is due to the large amount of residual air in the alveoli without stromal invasion, while mixed GGOs seen heterogeneous pattern, it is due to the presence of air and a solid component, when tumour invasion beyond the alveolar spaces which it happens when a GGO lesion develops a solid component.

In the pre-invasive lesions (*n* = 2) and in the minimally invasive adenocarcinomas (*n* = 5), the hyperechoic pattern seemed to be more predominant than that of the invasive adenocarcinoma (*n* = 7).

The different types of echogenicities could also be the result of artefacts.

Thus, after an initial learning curve performed in collaboration with expert radiologists, thoracic surgeons could be ready to use US for intraoperative identification of the GGOs.

The study needs further cases to corroborate our data. However, the searching of GGO was not performed only in surface of the lung. In fact, the probe used has a frequency of 12 MHz, and reaches a depth of about 15 cm.

## CONCLUSIONS

The results of our study indicate that IU could safely and effectively detect GGOs and define their features in terms of the size, depth, solid component and surgical margins, but that we were unable to differentiate the different subtypes of adenocarcinomas.

IU may thus assist surgeons to perform minimally invasive lung resections, with clear surgical margins, for the treatment of GGO; its characteristics of real-time feedback, repeatability, non-invasiveness and intraoperative ultrasonography provide valuable pathologic information based on morphological features such as the shape, edge, echo level, blood flow and acoustic shadow [24]. IU can certainly be used in robotic surgery, with a dedicated laparoscopic probe for the robot.

Working with ultrasound in daily clinical practice can help thoracic surgeons become familiar with US, before using it intraoperatively; it can lead to the quickly localization of the lesion by the pathologist.

The objective of our study aims to implement the use of intraoperative ultrasound as an alternative finger during VATS and therefore compensation in location and guidance for follow-up clinical decision guide.

### Limitations

The single-centre nature of the study limits the generalizability of the results. The study is a preliminary study that needs further cases to corroborate our data.

## Funding

PhD award University of Campania L. Vanvitelli.

**Conflict of interest: **None declared.

**Data availability**

All relevant data are within the manuscript and its Supporting Information files.

**Author contributions**

**Gaetana Messina:** Conceptualization; Writing—original draft. **Mary Bove:** Validation. **Giovanni Natale:** Formal analysis. **Antonio Noro:** Methodology. **Mario Martone:** Investigation. **Giorgia Opromolla:** Resources. **Vincenzo Di Filippo:** Resources. **Beatrice Leonardi:** Investigation. **Morena Fasano:** Data curation. **Rita Polito:** Data curation. **Alfonso Fiorelli:** Visualization. **Mario Santini:** Supervision. **Giovanni Vicidomini:** Project administration.

**Reviewer information**

Interactive CardioVascular and Thoracic Surgery thanks Emmanouil Ioannis Kapetanakis, Hitoshi Igai and the other anonymous reviewer(s) for their contribution to the peer review process of this article.

## ABBREVIATIONS

CT: Computed tomography
GGO: Ground-glass opacity
IU: Intraoperative ultrasound
